# Fatty Acid Profile, Volatile Organic Compound, and Physical Parameter Changes in Chicken Breast Meat Affected by Wooden Breast and White Striping Myopathies

**DOI:** 10.3390/ani13193136

**Published:** 2023-10-07

**Authors:** Eglė Lebednikaitė, Dovilė Klupšaitė, Elena Bartkienė, Jolita Klementavičiūtė, Ernestas Mockus, Lina Anskienė, Žana Balčiauskienė, Alius Pockevičius

**Affiliations:** 1 Department of Veterinary Pathobiology, Faculty of Veterinary Medicine, Lithuanian University of Health Sciences, 44307 Kaunas, Lithuania; alius.pockevicius@lsmu.lt; 2 Institute of Animal Rearing Technologies, Faculty of Animal Sciences, Lithuanian University of Health Sciences, 44307 Kaunas, Lithuania; dovile.klupsaite@lsmu.lt (D.K.); elena.bartkiene@lsmu.lt (E.B.); jolita.klementaviciute@lsmu.lt (J.K.); ernestas.mockus@lsmu.lt (E.M.); 3 Department of Animal Breeding, Faculty of Animal Sciences, Lithuanian University of Health Sciences, 44307 Kaunas, Lithuania; lina.anskiene@lsmu.lt; 4 Vilnius Department of the State Food and Veterinary Service, 08106 Vilnius, Lithuania; z.balciauskiene@kggroup.eu

**Keywords:** broiler, poultry meat quality, fatty acids, volatile compounds, hexanal, myopathy

## Abstract

**Simple Summary:**

Myopathies are muscle pathologies that affect fast-growing, heavy-weight broilers. The causes and mechanisms of the development of myopathies are still unclear. Broilers grow very quickly, their muscle fiber size increases rapidly, and the poor vascularization of muscle fibers leads to hypoxia. It is believed that hypoxia could be one of the main factors that induces the formation of myopathies. An elevated amount of alkyl aldehyde hexanal was detected in broilers’ breast muscles affected by myopathies. This finding may justify that oxygen deficiency could be an important factor in the development of broilers’ breast myopathies, as with a lack of oxygen, toxic substances are formed, and cell damage occurs. These substances damage cell lipids through chemical reactions, one of the main end products of which is aldehyde hexanal. Additionally, it was found that broilers’ breast meat affected by myopathies had a different flavor because myopathies affect the physical parameters and alter the fatty acid profile and volatile organic compound composition of the chicken breast muscle.

**Abstract:**

The aim of this research was to determine the impact of *pectoralis major* myopathies on the physical parameters, fatty acid (FA) profile, and volatile organic compound (VOC) composition of chicken breast meat. Samples were collected from *pectoralis major* of broilers with varying severity scores (normal, mild, and severe) of wooden breast (WB) and white striping (WS) myopathies. Chicken breast meat affected by severe myopathies expressed higher cooking loss, drip loss (*p* < 0.001), and yellowness (*p* < 0.05) compared to those of samples that were taken from broilers without myopathies (normal). The amount of monounsaturated fatty acids (MUFAs) was significantly higher in samples affected by mild and severe myopathies than in those without myopathies (*p* < 0.05). There was significantly more aldehyde hexanal in muscles affected by mild and severe myopathies than in muscles without myopathies (*p* < 0.05). In conclusion, WB and WS myopathies of the breast muscle not only affected the physical parameters of broiler meat but also may have influenced its FA profile and VOC composition. Additionally, an elevated amount of hexanal in muscles affected by WB together with WS suggests that oxidative stress could be important in the etiopathogenesis of WB and WS myopathies. Therefore, poultry meat affected by myopathies have the potential to alter breast meat flavor and composition.

## 1. Introduction

In 2022, the European Union produced 10.85 million metric tons of broiler meat [1]. Poultry meat is one of the most popular meats in the world due to its high protein and low-fat contents, a balanced n-6/n-3 polyunsaturated fatty acid (PUFA) ratio, low cholesterol, and the presence of various useful components [2,3]. Poultry specialists pushed biological boundaries to make modern broilers bigger and accelerate muscle growth in order to fulfill chicken meat demand [2,4]. Unfortunately, these improvements have also had unpleasant consequences. Myopathies of broiler muscles have alarmed the poultry industry [2]. The exact etiopathogenesis of novel wooden breast (WB) and white striping (WS) *pectoralis major* myopathies is still unclear [5,6,7]. It is believed that fast growth and enlarged breasts are the main predisposing factors of these muscle pathologies [8]. Increasing muscle fiber size leads to poor vascularization of muscle fibers, which leads to hypoxia and alters muscle metabolism (the delivery of essential nutrients and removal of waste products) [9,10]. Inadequate oxygen supply and limited metabolic waste removal leads to the accumulation of reactive oxygen species (ROS). This triggers oxidative stress, necrosis, and tissue degeneration [11,12,13]. It is known that degenerative processes and repair mechanisms take place in muscles affected by myopathies. Small hemorrhages, inflammation, fibrosis, and lipidosis are established in these muscles [5,8]. Lipids, especially phospholipids, make up the cell membrane. The proportion of essential PUFAs in phospholipids is considerable (over 30%). Phospholipids with a higher PUFA concentration are crucial for the development of meat flavor. Furthermore, as a result of a higher concentration of PUFAs, they are more susceptible to oxidation compared to triacylglycerides [14,15]. It is known that oxidative stress can induce lipid peroxidation, which is involved in various pathological states whose etiologies are still unclear [16]. Hydroperoxides are primary lipid oxidation products. Secondary PUFA peroxidation products are volatile organic compounds (VOCs), mostly aldehydes, such as malondialdehyde (MDA), hexanal, and 4-hydroxynonenal [17,18,19,20]. VOCs are substances that provide information about the quality of food and are also involved in aroma formation [21]. Many factors influence the quality of poultry: sex, strain, age, environmental factors, exercise, diet, and processing practices mainly focused on chilling, debonding time, marination, and electrical stunning [22,23,24,25]. Meat affected by myopathy is sometimes downgraded or even rejected for human consumption, which leads to economic losses for the poultry industry [5,26]. It is known that myopathies can also influence the taste, texture, and physical parameters of meat [27,28,29]. However, only a few studies have been conducted regarding VOCs in raw chicken meat, especially with myopathies [30,31]. Therefore, the aim of this research was to determine the effect of *pectoralis major* myopathies not only on the physical parameters but also on the FA profile and VOC composition of chicken breast meat.

## 2. Materials and Methods

### 2.1. Ethical Considerations

This study did not require consent or ethical approval according to the European Directive 2010/63/EU. The animals were slaughtered in strict accordance with European slaughter regulations (CE n° 1099/2009 of 24 September 2009) for the protection of animals at the time of killing (Ref. *Official Journal of the European Union L 303/1*). Permission to obtain the samples was granted by the management and veterinarian of the slaughterhouses before the study commenced.

### 2.2. Sample Collection

Eighteen samples were taken from the *pectoralis major* of Ross 308 (male and female) broilers in a slaughterhouse. All chickens were bred and raised in a traditional intensive system without antibiotics. They were slaughtered according to standard industrial practice at 41 days old, with an average live weight of 2.5 kg. The muscle samples were collected without bone and skin from broilers with varying WB and WS myopathies. All samples were classified according to the degree of severity. The severity of WB and WS myopathies was established visually and via palpation of the *pectoralis major*: normal (*n* = 6): no pale areas, no hardness, and no thick liquid or white lines of breast fillet; mild (*n* = 6): hardness and pale muscle only in the cranial part of the breast fillet, light-yellow viscous liquid on the breast fillet, and up to 1 mm thick white lines are visible; severe (*n* = 6): hardness, pale areas throughout the breast fillet, a lot of light-yellow, thick, and viscous liquid on the breast fillet, and white lines clearly visible, thicker than 1 mm. White striping myopathy classification criteria were described according to Kuttappan et al. [32].

After being classified and collected, the samples were weighed individually (each sample weighed about 300 g) and packed in identical freezer bags of the same batch. A sticker with a number was attached onto each sample, and the samples were transported to a laboratory under refrigeration conditions (±4 °C). The samples were frozen (–20 °C) until further analysis. Physical, FA profile, and VOC analyses were performed on all collected chicken breast samples.

### 2.3. Physical Analysis

The technological meat properties were analyzed in the manner described by Rozanski et al. [33] and AOAC [34]. Muscle pH was measured with a pH meter (model Inolab 3, Hanna Instruments, Italy) calibrated to pH 4.0 and 7.0.

The whole *pectoralis major* was used to perform the quality evaluation. According to Klupsaite et al. [35], water-holding capacity, drip loss, cooking loss, and shear force were determined. The filter paper press method was used to determine water-holding capacity. The sample (2 g) was placed on filter paper (Whatman filter paper 41/ashless), compressed between two plexiglass sheets (10 × 10 cm), and received pressure exerted by a weight of 1 kg for 10 min. Drip loss was measured as the weight lost during the suspension of a standardized (40–50 g and approximately 30 × 60 × 25 mm) *pectoralis major* sample (in an airtight container over 24 h at 4 °C). Cooking loss was established as raw to cooked and then shear force was conducted on cooked meat. The weight difference of a sample (in a plastic container) before and after cooking in a water bath (internal temperature of 70 °C for 30 min) was used to calculate cooking loss. For the evaluation of shear force, three cylindrical samples with a diameter of 1.27 cm were removed from each sample that was used to determine cooking loss. TA-XT Plus texture analyzer (TA.XT plus Texture Analyzer, Stable Microsystems Ltd., Surrey, UK) coupled to a Warner–Bratzler device was used to measure shear force. After oven-drying the samples at 105 °C for 24 h, the dry matter content of the muscles was determined.

Meat color (lightness, redness, and yellowness) was measured after a 30–40 min blooming and remeasured in the midday using a Minolta Chroma Meter colorimeter (CR-400, Minolta Camera, Osaka, Japan) with a closed cone, set on the lightness, redness, and yellowness systems. The chromameter was calibrated with a white tile (Y = 92.8, x = 0.3160, y = 0.3323) using Illuminant D-65, a 2° standard observer and an 8 mm aperture. The color was measured in the middle part of the *pectoralis major*. Before measurement, it was cut in two parts, and the cut place was measured.

### 2.4. Chemical Analysis

#### 2.4.1. Fatty Acid Content

*Pectoralis major* was minced before the chemical analysis. The extraction of lipids for fatty acid analysis was performed with chloroform/methanol (2:1 *v*/*v*) as described by Pérez-Palacios et al. [36]. Further analysis was performed following the procedures outlined in Klupsaite et al. [35]. A GC-2010 Plus gas chromatograph (Shimadzu Corporation, Kyoto, Japan) together with a GCMS-QP2010 mass spectrometer (Shimadzu Corporation, Kyoto, Japan) were used to analyze the fatty acid composition. A calibration curve was used to measure the concentration of fatty acid methyl esters (FAME), and the results were represented as a percentage of the total concentration of FAME in the sample.

#### 2.4.2. Malondialdehyde (MDA)

MDA was analyzed according to the method described by Mendes et al. [37] with some modifications outlined in Klupsaite et al. [35]. In the sample, MDA was derived with thiobarbituric acid solution. Varian ProStar HPLC system (Varian Corp., Palo Alto, CA, USA) was used for chromatographic analysis.

#### 2.4.3. Volatile Organic Compound (VOC) Profile

The VOC of poultry meat samples were analyzed using headspace solid-phase microextraction (HS-SPME) coupled to gas chromatography-mass spectrometry (GC-MS) A solid-phase microextraction (SPME) device with Stableflex (TM) fiber-coated with a 50 µm DVB-PDMS-Carboxen™ layer (Supelco, USA) was used to prepare the samples. For headspace extraction, 4 g of the homogenized sample was transferred to a 20 mL extraction vial. It was sealed with polytetrafluoroethylene septa and thermostated at 60 °C for 30 min. The fiber was exposed to the headspace of the vial for 30 min. Desorption time was 2 min. The prepared samples were analyzed with a GCMS-QP2010 (Shimadzu, Japan) gas chromatograph and a mass spectrometer. A Stabilwax-Da capillary column (30 m × 0.25 mmID × 0.25 µm film thickness) was used for the analysis. The mass spectrometer operated at full scan mode (35–500 *m*/*z*). The following conditions were used for the analysis: column flow rate (helium gas, 99.999% purity) of 0.65 mL/min, injector temperature of 250 °C, ion source temperature of 220 °C, and interface temperature of 280 °C. The temperature gradient was programmed to start at 40 °C (3 min hold) and rise to 250 °C (5 °C/min) (5 min hold). The volatile organic compounds were identified according to the mass spectra libraries (NIST11, NIST11S, and FFNSC2).

### 2.5. Statistical Analysis

The results were calculated using SPSS version 29.0 (IBM Corp., Armonk, New York, NY, USA) and are presented as the means and standard deviations (SD). The data were analyzed using one-way ANOVA, and a post hoc Tukey test was used to compare differences in the means between the groups. A *p*-value of less than 0.05 was considered to indicate statistical significance.

## 3. Results

Data for dry matter, drip loss, pH, water-holding capacity, cooking loss, and shear force can be found in Table 1. Samples affected by severe myopathies contained 6.14% lower dry matter than normal samples of muscles (*p* ≤ 0.01) and 5.28% lower than samples of muscles affected by mild myopathies (*p* ≤ 0.05).

Drip loss in samples of muscles affected by severe myopathies was 55.09% higher compared to normal samples without myopathies (*p* ≤ 0.01).

Cooking loss in the muscles affected by severe myopathies was 68.40% higher compared to muscle without myopathies (*p* ≤ 0.001) and 38.43% higher compared to muscles affected by mild myopathies (*p* ≤ 0.001).

However, there was no effect of the myopathies’ severity on shear force, pH, or water-holding capacity (*p* > 0.05).

Table 2 shows that the yellowness of the muscle samples affected by severe myopathies was 20.09% higher compared to that of samples without myopathies (normal) (*p* ≤ 0.05). Additionally, the yellowness of the muscle samples affected by mild myopathies was 27.76% higher compared to normal samples without myopathies (*p* ≤ 0.01).

However, the severity of myopathies did not have a significant effect on the lightness and redness of chicken breast meat (*p* > 0.05).

Table 3 shows that overall, the amount of monounsaturated fatty acids (MUFAs) was 5.09% higher in muscle affected by severe myopathies compared to that in muscle without myopathies (normal) (*p* < 0.05). Additionally, the amount of MUFAs was 5.45% higher in muscle affected by mild myopathies compared to that in muscle without myopathies (normal) (*p* < 0.05).

Among the investigated saturated fatty acids (SFAs), the amount of myristic acid was 6.5-fold higher in severe myopathy samples compared to that in samples without myopathies (*p* ≤ 0.01). However, the amount of stearic acid was 17.81% lower in muscle affected by severe myopathies compared to that in muscle without myopathies (*p* ≤ 0.05). Additionally, the amount of stearic acid was 16.04% lower in muscle with mild myopathies compared to that in muscle without myopathies (*p* ≤ 0.05).

Among MUFAs, the amount of oleic acid was 4.73% higher in muscles affected by severe myopathies compared to that in normal muscles without myopathies (*p* ≤ 0.05). Furthermore, the amount of palmitoleic acid was 30.48% higher in muscles affected by mild myopathies compared to that in normal muscle without myopathies (*p* ≤ 0.05). Other fatty acid contents were unaltered (*p* > 0.05) in muscles affected by myopathies compared to those in muscles without myopathies.

**Figure 1:**
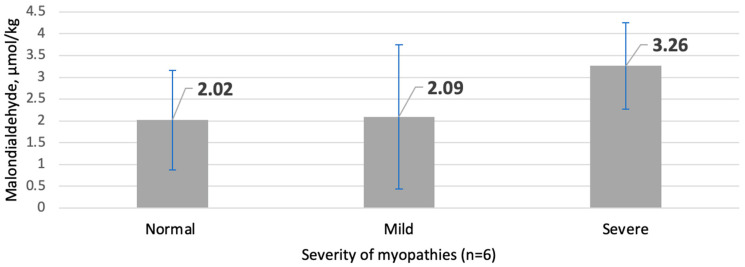
Means and standard deviations of malondialdehyde (MDA) content in *pectoralis major* of broilers with and without myopathies.

According to the concentration of MDA (µmol/kg), as seen in Figure 1, severe myopathies expressed 61.39% higher concentrations than the control samples of muscles; however, the differences between the means were not statistically significant (*p* > 0.05).

VOC analysis identified 22 individual substances: aldehydes (11), alcohols (8), esters (2), and furans (1). Amounts of VOC (area percentage according to the identified compounds) in the *pectoralis major* of Ross 308 broilers affected by myopathies are shown in Table 4. The hexanal amount was 33.76% higher in muscles affected by severe myopathies (*p* ≤ 0.01) and 26.21% higher in muscles affected by mild myopathies (*p* ≤ 0.05) compared to that in muscles without myopathies (normal).

The amount of allyl 2-ethylbutyrate was 4.3-fold higher in muscles affected by severe myopathies (*p* ≤ 0.01) and 4.2-fold higher in mild myopathies muscles (*p* ≤ 0.01) compared to that in muscles without myopathies (normal). Compared to those in muscles without myopathies (normal), the amounts of 2-octenal, 2-octen-1-ol, and 2-decenal were 9.6-fold (*p* ≤ 0.01), 2.2-fold (*p* ≤ 0.05), and 7.9-fold higher (*p* ≤ 0.01), respectively, in muscles affected by severe myopathies. Furthermore, the amount of 2-decenal was approximately 3.8 times higher in muscles affected by severe myopathies compared to that in muscles affected by only mild myopathies (*p* ≤ 0.05).

However, 2-ethyl-1-hexanol and benzaldehyde showed an opposite tendency, and their amounts were 77.12% (*p* ≤ 0.05) and 68.64% (*p* ≤ 0.05) lower, respectively, in muscles affected by severe myopathies compared to those in muscles without myopathies (normal). Other substance amounts were unaltered (*p* > 0.05) in muscles affected by myopathies compared to those in muscles without myopathies.

## 4. Discussion

According to the results, chicken breast meat affected by severe myopathies showed higher cooking loss and drip loss compared to those of samples taken from broilers without myopathies (normal). Increasing cooking loss results in less juicy and less tender meat [38]. Regarding drip loss, water escapes from raw poultry meat during storage. Oxidative stress can damage cell membranes. Therefore, water can escape, resulting in higher drip loss as well as cooking loss [39]. This study’s results corroborate those of a previous study by Wang et al. [40]. Cooking loss and drip loss were greater in breast meat affected by severe myopathies compared to those of breast meat without myopathies (normal). Furthermore, according to other authors [41,42], a mild degree of these combined myopathies does not significantly affect drip loss, but severe WS or WB myopathies significantly increase drip loss in *pectoralis major* samples. However, according to Dalgaard et al. [41], even mild degree of WB were associated with increased cooking loss. We hypothesized that these differences could be due to the bird’s age, weight, and cooking procedure.

According to this study, the dry matter content was significantly lower in *pectoralis major* samples affected by severe myopathies. According to the literature, muscle fiber necrosis, degeneration, edema, and inflammatory processes are observed in histopathological muscle samples affected by myopathies [5,8]. We strongly believe that these observations could be related to a lower dry mass content in the muscle affected by WB and WS myopathies due to the loss of muscle tissues and accretion of extracellular water as a result of edema [43,44].

According to the results, yellowness was significantly higher in samples affected by mild or severe myopathies compared to that in normal samples without myopathies. Wang et al. [40] also reported higher yellowness in breast muscle with myopathies when severe WB is present. According to the literature, WB myopathy is characterized by pale areas, translucent fluid, or citrine coloration on the surface of *pectoralis major* due to polyphasic muscle fiber degeneration and fibrosis, as well as an accumulation of intramuscular fat [5]. We believe that these pathomorphological changes of muscle could affect the color parameters of the *pectoralis major* of broilers and induce the color change to more yellowness.

Overall, palmitic, stearic, oleic, and linoleic acids were found to be the main fatty acids of chicken breast meat. However, palmitic and linoleic FA amounts were comparable in meat affected and not affected by myopathies. Additionally, compared to those in normal samples, *pectoralis major* samples affected by severe myopathies had higher levels of MUFAs, including oleic and palmitoleic acids. Similar results were found by Liu et al. [45], who observed higher levels of MUFAs as well as lower levels of SFAs and PUFAs in WB/WB+WS affected meat. Soglia et al. [46] and Oliveira et al. [47] found no significant differences in PUFA and MUFA contents between normal breast muscle without myopathies and breast muscle affected by WB and WS myopathies. However, Soglia et al. [46] found higher SFA in breasts with WS myopathy but not with WB and WB+WS myopathies. Changes in the FA profile of chicken broiler meat may support the theory that oxidative stress is important in the etiopathogenesis of broiler myopathies.

VOCs are substances that provide information about the quality of food and are involved in aroma formation [21]. Those substances are formed during the Maillard reaction, Strecker degradation, lipid oxidation, degradation of thiamine, carbohydrate degradation, as well as interactions between reaction products [48,49,50,51,52]. Aldehydes are secondary products of fatty acid oxidation [53]. According to the literature, aldehydes are better indicators of lipid oxidation than other volatile substances [54]. In this study, aldehydes were the largest established VOC group in broiler meat affected by WB together with WS. Among all established aldehydes, hexanal showed the highest amount in the studied samples; this aldehyde was also significantly more abundant in muscles affected by WB together with WS. Filho et al. [55] announced that raw and cooked WS meat showed higher hexanal concentrations than samples of muscles without myopathy. Additionally, hexanal is mainly derived from the oxidation of linoleic and oleic acids [30,55,56]. According to our research, higher levels of oleic acid were also established in this study, and this may explain elevated concentrations of hexanal in WB- and WS-affected meat. Additionally, according to the literature, hexanal is associated with the pleasant aroma of grass and provides a green and fatty character to different meat species [30,56]. Nevertheless, hexanal, as a secondary oxidation product, may cause a negative smell and taste as well as loss of color and nutritional value. It is also associated with off-odors due to its effect on lipids, proteins, and other substances [57,58,59,60]. Other aldehydes, including 2-decenal and 2-octenal, were also significantly more abundant in muscles affected by myopathies. However, benzaldehyde showed the opposite tendency—less of it was found in muscles affected by severe WB and WS myopathies.

The second most prevalent established group was alcohol. Alcohols also mainly originate from the oxidative decomposition of lipids [61]. There was more 2-octen-1-ol in muscles affected by WB together with WS. However, 2-ethyl-1-hexanol showed the opposite tendency—there was less of it in muscles affected by severe myopathies. Generally, raw meat is weakly flavored, and volatile substances are generated during heating, so for those reactions to occur, cooking temperatures are needed. According to the literature, the amount of VOCs in meat increases significantly after cooking [31,62]. Finally, according to this study, WB together with WS influenced the VOCs of chicken breast meat and its flavor, but more research is still needed to evaluate the effect of cooking on VOCs in WB- and WS-affected breast meat because heat can induce the reactions necessary for VOC formation.

## 5. Conclusions

Chicken breast meat affected by WB and WS myopathies showed higher yellowness, cooking loss, and drip loss compared to broiler meat without myopathies. Furthermore, meat affected by myopathies had a lower dry matter content. WB and WS myopathies have also influenced the FA and VOC profiles of chicken breast meat. Aldehyde hexanal, which is one of the main lipid peroxidation products, was significantly more abundant in the *pectoralis major* affected by myopathies. This result may justify that oxidative stress is important in the etiopathogenesis of WB and WS myopathies. Therefore, it may be predicted that myopathies could impair the flavor of broiler breast meat.

## Figures and Tables

**Table 1:** pH, dry matter content, drip loss, water-holding capacity, cooking loss, and shear force of *pectoralis major* of broilers affected by myopathies (mean ± SD).

Variables	Severity of Myopathies			*p*-Value
	Normal ^a^ (*n* = 6)	Mild ^b^ (*n* = 6)	Severe ^c^ (*n* = 6)	
pH	6.05 ± 0.11	6.04 ± 0.16	6.03 ± 0.11	0.942
Dry matter content, %	26.38 ± 0.74 ^c^	26.14 ± 0.56 ^c^	24.76 ± 0.88 ^a,b^	0.004
Drip loss, %	2.85 ± 0.82 ^c^	3.63 ± 0.59	4.42 ± 0.44 ^a^	0.003
Water-holding capacity, %	65.21 ± 1.67	65.66 ± 1.59	64.45 ± 1.99	0.498
Cooking loss, %	12.47 ± 1.29 ^c^	15.17 ± 2.95 ^c^	21.00 ± 1.57 ^a,b^	<0.001
Shear force, kg/cm^2^	1.64 ± 0.32	1.48 ± 0.34	1.49 ± 0.50	0.731

Superscripts ^a, b, c^ assigned to each group of the myopathies’ severity indicate comparison group when statistically significant mean differences were found (*p* < 0.05).

**Table 2:** Color parameters of *pectoralis major* of broilers affected by myopathies (mean ± SD).

Variables	Severity of Myopathies			*p*-Value
	Normal ^a^ (*n* = 6)	Mild ^b^ (*n* = 6)	Severe ^c^ (*n* = 6)	
Lightness, NBS	51.22 ± 5.75	52.97 ± 3.19	52.45 ± 2.77	0.755
Redness, NBS	11.61 ± 1.13	10.05 ± 1.26	10.81 ± 1.69	0.180
Yellowness, NBS	8.61 ± 1.75 ^b,c^	11.00 ± 0.55 ^a^	10.34 ± 0.77 ^a^	0.008

Superscripts ^a, b, c^ assigned to each group of the myopathies’ severity indicate comparison group when statistically significant mean differences were found (*p* < 0.05).

**Table 3:** Fatty acid composition (percentage of total fatty acids) of *pectoralis major* of broilers affected by myopathies (mean ± SD).

Fatty Acid	Nomenclature	Severity of Myopathies			*p*-Value
		Normal ^a^ (*n* = 6)	Mild ^b^ (*n* = 6)	Severe ^c^ (*n* = 6)	
Myristic acid	C14:0	0.04 ± 0.01 ^c^	0.20 ± 0.14	0.26 ± 0.13 ^a^	0.011
Palmitic acid	C16:0	19.91 ± 1.02	19.82 ± 0.56	20.15 ± 1.28	0.838
Stearic acid	C18:0	9.04 ± 0.76 ^b,c^	7.59 ± 0.98 ^a^	7.43 ± 1.12 ^a^	0.020
SFA		28.99 ± 1.65	27.61 ± 1.35	27.84 ± 1.84	0.316
Palmitoleic acid	C16:1	2.46 ± 0.40 ^b^	3.21 ± 0.49 ^a^	2.82 ± 0.48	0.040
Oleic acid	C18:1	30.84 ± 0.67 ^c^	32.05 ± 0.77	32.30 ± 1.04 ^a^	0.020
Gondoic acid	C20:1 ω9	0.45 ± 0.12	0.34 ± 0.06	0.36 ± 0.03	0.073
MUFA		33.76 ± 0.92 ^b,c^	35.60 ± 1.14 ^a^	35.48 ± 1.27 ^a^	0.020
Linoleic acid	C18:2 ω6	34.00 ± 1.50	34.09 ± 1.36	33.94 ± 1.60	0.984
γ-Linolenic	C18:2 ω6	0.04 ± 0.02	0.05 ± 0.03	0.07 ± 0.03	0.165
α-Linolenic acid	C18:3 α ω3	2.50 ± 0.53	2.01 ± 0.21	2.03 ± 0.23	0.055
Eicosadienoic acid	C20:2 ω6	0.35 ± 0.08	0.31 ± 0.06	0.29 ± 0.11	0.424
Arachidonic acid	C20:4 ω6	0.36 ± 0.11	0.32 ± 0.10	0.35 ± 0.12	0.807
PUFA		37.26 ± 1.79	36.79 ± 1.48	36.68 ± 1.54	0.807
Omega 6		34.76 ± 1.54	34.78 ± 1.37	34.64 ± 1.55	0.986
Omega 3		2.50 ± 0.53	2.01 ± 0.21	2.03 ± 0.23	0.055

Superscripts ^a, b, c^ assigned to each group of the myopathies’ severity indicate comparison group when statistically significant mean differences were found (*p* < 0.05).

**Table 4:** Amounts of volatile compounds (area percentage according to the identified compounds) in *pectoralis major* of broilers affected by myopathies (mean ± SD).

Volatile Compounds	Severity of Myopathies			*p*-Value
	Normal ^a^ (*n* = 6)	Mild ^b^ (*n* = 6)	Severe ^c^ (*n* = 6)	
Aldehydes				
Hexanal	27.58 ± 5.26 ^b,c^	34.81 ± 2.69 ^a^	36.89 ± 2.95 ^a^	0.002
Heptanal	0.67 ± 0.88	1.09 ± 1.05	0.99 ± 0.74	0.706
Octanal	5.38 ± 2.87	5.85 ± 0.80	5.16 ± 0.59	0.789
Nonanal	13.62 ± 4.07	13.43 ± 2.63	13.59 ± 1.84	0.993
2-Octenal	0.15 ± 0.38 ^c^	0.69 ± 0.81	1.45 ± 0.11 ^a^	0.002
Decanal	nd	0.36 ± 0.87	0.90 ± 1.03	0.163
Benzaldehyde	19.93 ± 12.64 ^c^	11.67 ± 5.52	6.25 ± 2.93 ^a^	0.033
2-Decenal	0.13 ± 0.33 ^c^	0.27 ± 0.66 ^c^	1.03 ± 0.24 ^a,b^	0.007
2,4-Dodecadienal	nd	nd	0.25 ± 0.39	0.117
2-Dodecenal	0.10 ± 0.25	nd ^c^	0.45 ± 0.38 ^b^	0.023
Alcohols				
1-Pentanol	2.97 ± 1.11	3.07 ± 0.44	3.33 ± 0.31	0.677
1-Hexanol	0.27 ± 0.42	0.46 ± 0.50	1.03 ± 0.60	0.053
1-Octen-3-ol	9.51 ± 7.44	14.18 ± 1.18	14.98 ± 1.35	0.101
1-Heptanol	3.26 ± 1.00	2.90 ± 0.33	3.19 ± 0.32	0.605
2-Ethyl-1-hexanol	2.36 ± 1.70 ^c^	1.32 ± 0.51	0.54 ± 0.34 ^a^	0.027
1-Octanol	6.70 ± 2.74	5.02 ± 0.76	4.87 ± 0.35	0.137
2-Octen-1-ol	1.09 ± 0.87 ^c^	1.70 ± 0.63	2.41 ± 0.47 ^a^	0.015
Benzyl-alcohol	5.19 ± 3.54	2.81 ± 1.60	1.78 ± 1.21	0.065
Esters				
n-Caproic acid vinyl ester	0.77 ± 1.89	nd	0.40 ± 0.98	0.565
Allyyl-2-ethylbutyrate	0.09 ± 0.13 ^b,c^	0.38 ± 0.20 ^a^	0.39 ± 0.08 ^a^	0.003
Furans				
2-Pentylfuran	0.12 ± 0.29	nd	nd	0.391
Others				
1-Tetradecyne	0.12 ± 0.29	nd	0.13 ± 0.33	0.614

Superscripts ^a, b, c^ assigned to each group of the myopathies’ severity indicate comparison group when statistically significant mean differences were found (*p* < 0.05); nd = not detected.

## Data Availability

Not applicable.
